# Invasive Mediastinal Aspergillosis in an 18-Year-Old Immunocompetent Female Leading to Stroke

**DOI:** 10.7759/cureus.66014

**Published:** 2024-08-02

**Authors:** Muhammad Umar Mian, Muhammad Mehwar Anjum, Hassan Abdullah, Saad Nadeem, Rashid N Siddiqui

**Affiliations:** 1 Internal Medicine, Allama Iqbal Medical College, Lahore, PAK; 2 Internal Medicine, Sheikh Zayed Medical College, Rahim Yar Khan, PAK; 3 Internal Medicine, Ittefaq Hospital, Lahore, PAK

**Keywords:** antifungal therapy, timely diagnosis, fungal stroke, cardiac aspergillosis, eosinophilia, immunocompetent patient, mediastinal aspergillosis, invasive pulmonary aspergillosis

## Abstract

We present a case of invasive pulmonary aspergillosis in an immunocompetent young female. An 18-year-old female presented with symptoms of a left-sided middle cerebral artery (MCA) stroke with right arm weakness and aphasia. Computed tomography (CT) brain confirmed the diagnosis of stroke. Further history revealed that the patient had been experiencing low-grade fevers with occasional shortness of breath for the past year. The blood work had eosinophilia at that time for which she was given mebendazole but saw little improvement. Chest X-rays showed upper lobe consolidation for which a tuberculosis (TB) workup was also done, which also came out negative. At the current presentation, she underwent further workup with echocardiography and eventual ultrasound-guided mediastinal biopsy that ultimately led to the correct diagnosis of aspergillosis. However, sadly, it was already too late for the patient who passed away one day after the commencement of the amphotericin B therapy. This paper hopes to decrease the threshold of clinical suspicion for invasive aspergillosis (IA) regardless of the immunity status of the patient, especially if they are presenting with an unrelenting mediastinal or pulmonary symptom complex in the setting of eosinophilia.

## Introduction

Invasive aspergillosis (IA) is a condition that primarily affects individuals with weakened immune systems and tends to primarily target the lungs, although it can spread to other parts of the body as well, including the sinuses, gastrointestinal (GI) tract and brain, and is a common cause of fungal endocarditis second only to *Candida *[1]. The fungus can lead to opportunistic infections in three major types: allergic bronchopulmonary aspergillosis, aspergilloma, and IA. Instances of IA in the mediastinum of an immunocompetent patient have been rarely documented [2]. Here, we present a case of an 18-year-old female patient who presented with symptoms of stroke and also had a one-year history of occasional dyspnea.

## Case presentation

An 18-year-old unmarried female resident of Lahore presented to Ittefaq Hospital Trust, Lahore, Pakistan, in March 2024, with right-sided weakness and aphasia for two days.

The history of the patient dates back to around one year ago when she developed an intermittent low-grade fever, without rigors or chills, relieved by antipyretics, associated with fatigue and occasional shortness of breath on exertion. She also had undocumented weight loss over the course of this illness.

Before the current illness, she had never remained admitted for any ailment in her life and had completed her vaccination schedule, and her parents did not recall any severe respiratory infection in her childhood.

Her baseline investigations at the time were positive only for eosinophilia, and subsequently, she was prescribed a course of mebendazole 100 mg twice daily for three days for empiric treatment of worm infestation, which is prevalent in the region.

Her symptoms subsequently improved for a few months but returned with similar complaints of fever and shortness of breath along with numbness on the right side of the body.

Further workup revealed iron deficiency anemia with transferrin saturation of 11%, which was managed with oral iron supplementation of polysaccharide iron complex 150 mg once daily. She did complain of occasional menorrhagia, had normal bowel habits, and had a normal urinalysis showing no microscopic hematuria.

Besides the medications listed above, she was on no regular medication that would otherwise explain the eosinophilia. The family did not recall any episode suggesting allergy or anaphylaxis to any food component or drug.

**Figure 1:**
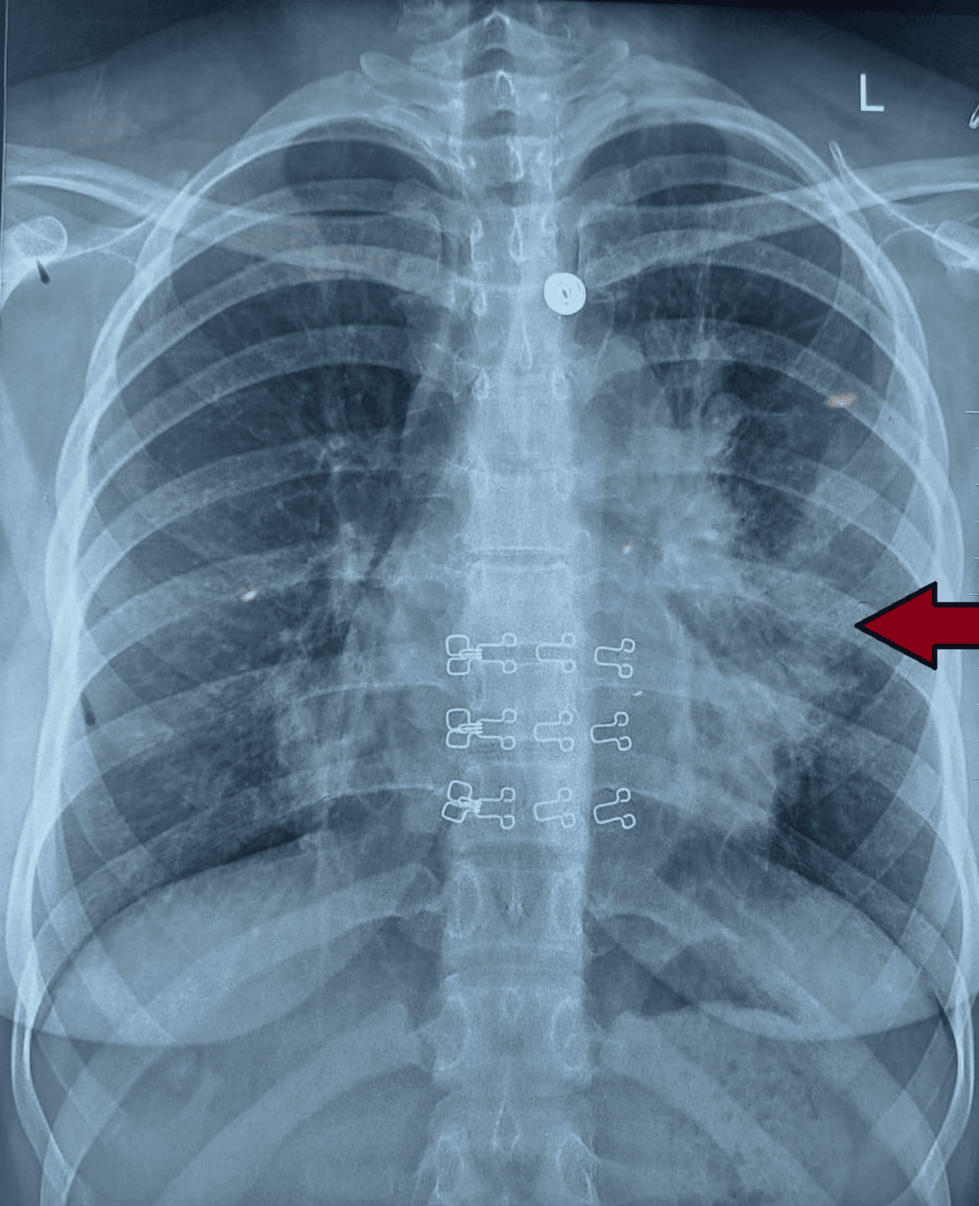
Chest X-ray showing left-sided consolidatory changes

As her shortness of breath continued to worsen despite these measures, she got a chest X-ray that showed left-sided consolidatory changes for which an acid-fast bacillus (AFB) smear and Gene Xpert were done, which also came out to be normal (Figure 1).

Finally, she presented to our facility with aphasia and dense right-sided hemiplegia.

**Figure 2:**
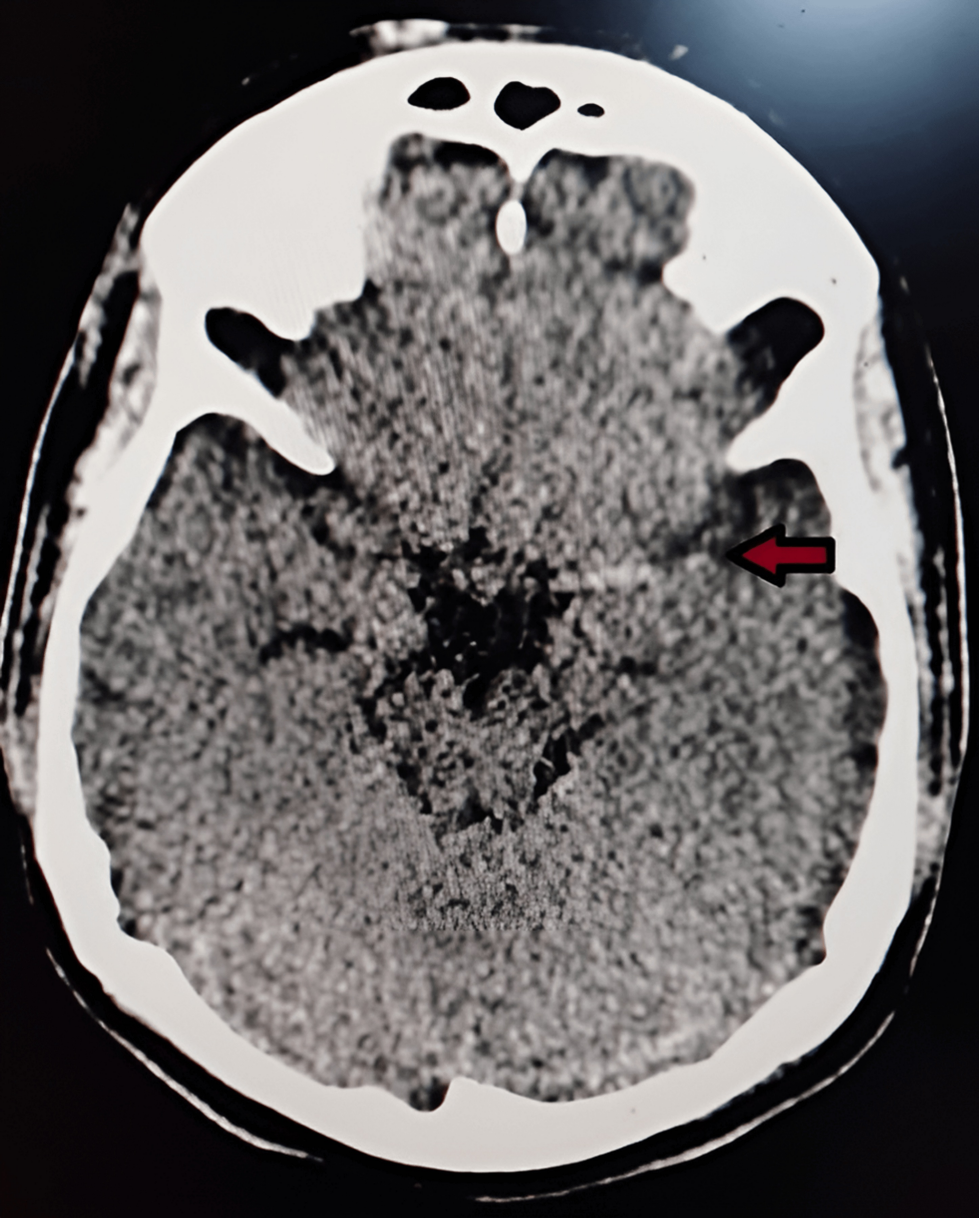
CT brain with dense left middle cerebral artery (MCA) sign

Her computed tomography (CT) scan of the brain showed bilateral basal ganglia calcification and a hyperdense left middle cerebral artery (MCA) sign on current admission (Figure 2).

Her blood work during the current admission showed normocytic anemia with normal B12 and folate levels, transferrin saturation 28%, negative antinuclear antibody (ANA) and extractable nuclear antigen (ENA) profile, negative antineutrophil cytoplasmic antibody (ANCA) studies, IgE levels of 9,126 IU/ml, erythrocyte sedimentation rate (ESR) of 97 mm/hour, and C-reactive protein (CRP) of 56 mg/L (Table 1). Her clotting profile, liver function tests, and renal function tests were also unremarkable.

**Table 1:** Lab reports showing marked eosinophilia, anemia of chronic disease, and increased serum IgE levels

Test	Reference range	Result
Iron studies		
Serum iron level	37-145 ug/dL	24.0
TIBC	274-385 ug/dL	208
Serum ferritin	4.63-204 ng/mL	288.4
CBC		
WBC	4-11 x 10^3^/uL	7.6
RBC	4-5.2 x 10^6^/uL	4.05
Hemoglobin	11.5-16 g/dL	9.2
Hematocrit	34-45%	31.0
MCV	79-95 fL	76.0
MCH	26-32 pg	23.0
MCHC	32-36 g/dL	30.0
RDW-CV	11.5-14.5%	15.4
Platelets	150-400 x 10^3^/uL	269.0
MPV	7.2-13 fL	8.7
Neutrophils	34-70%	50.0
Lymphocytes	19-52%	20.0
Monocytes	2-12%	5.0
Eosinophils	1-6%	25.0
Reticulocyte count	0.5-1.5%	0.5
ESR	0-20 mm/hour	77
Special chemistry		
IgE	<100 IU/mL	9126
Serum LDH	25-220 U/L	130.0
Serum B12	197-771 ng/mL	>2000
Serum folate	3.1-20.5 ng/mL	9.3
HbA1c	4-6.5 %	5.7
Urinalysis		
Color	Yellow	Yellow
Appearance	Clear	Clear
Specific gravity	1.005-1.030	1.01
pH	4.6-8	6.0
Protein	Negative	Negative
Glucose	Negative	Negative
Ketones	Negative	Negative
Bilirubin	Negative	Negative
RBC	0-2 /HPF	0-2
WBC	0-2 /HPF	Nil
Epithelial cells	0-2 /HPF	0-2
Crystals	Nil /HPF	Nil
Casts	Nil /HPF	Nil

**Figure 3:**
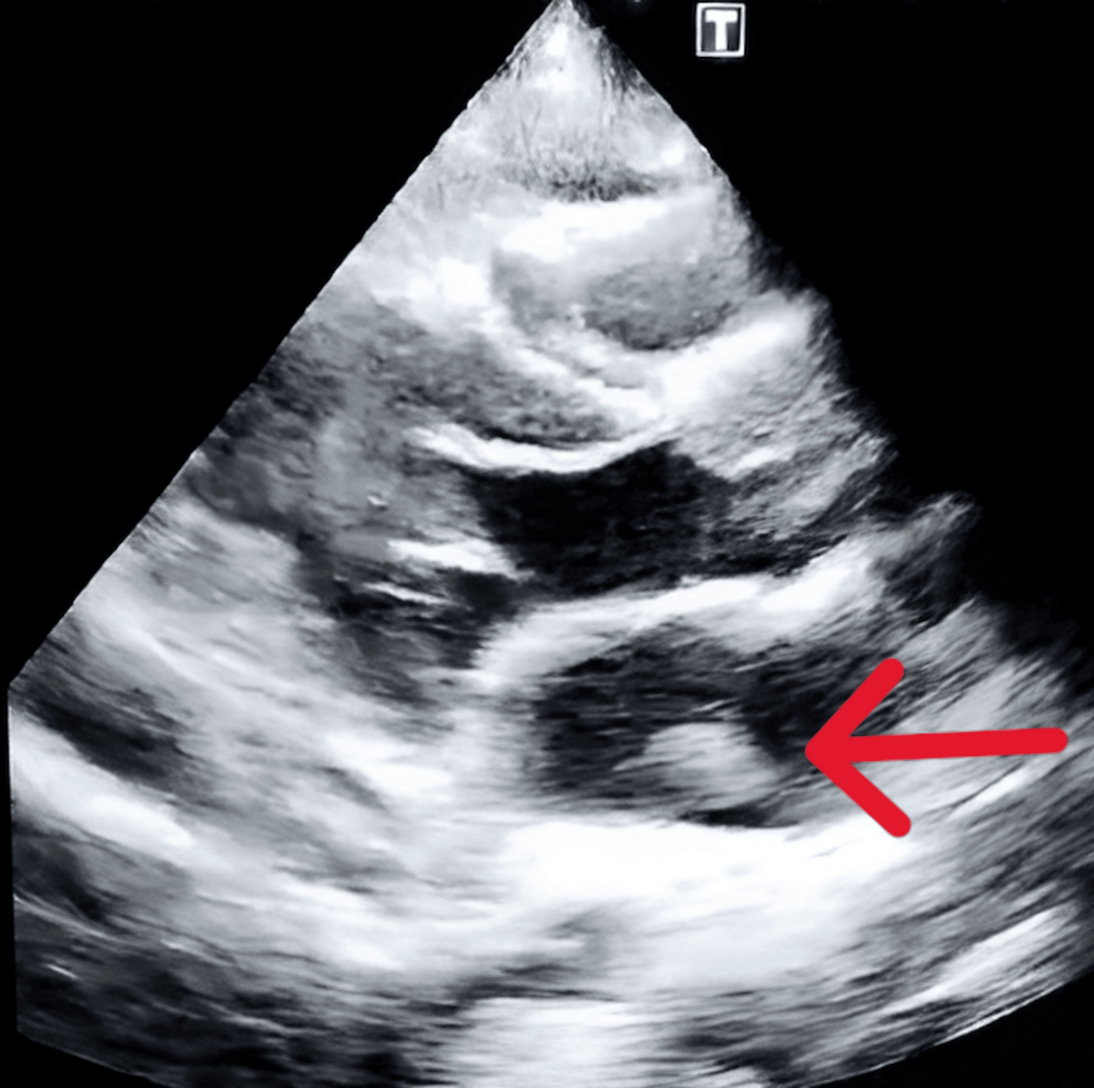
Echocardiogram showing a mobile oscillating mass measuring 11 x 15 mm in the left atrium

A transthoracic echocardiogram revealed a mobile oscillating mass in the left atrium measuring 11 × 15 mm attached to the left atrial free wall (Figure 3). The left ventricular function was normal.

**Figure 4:**
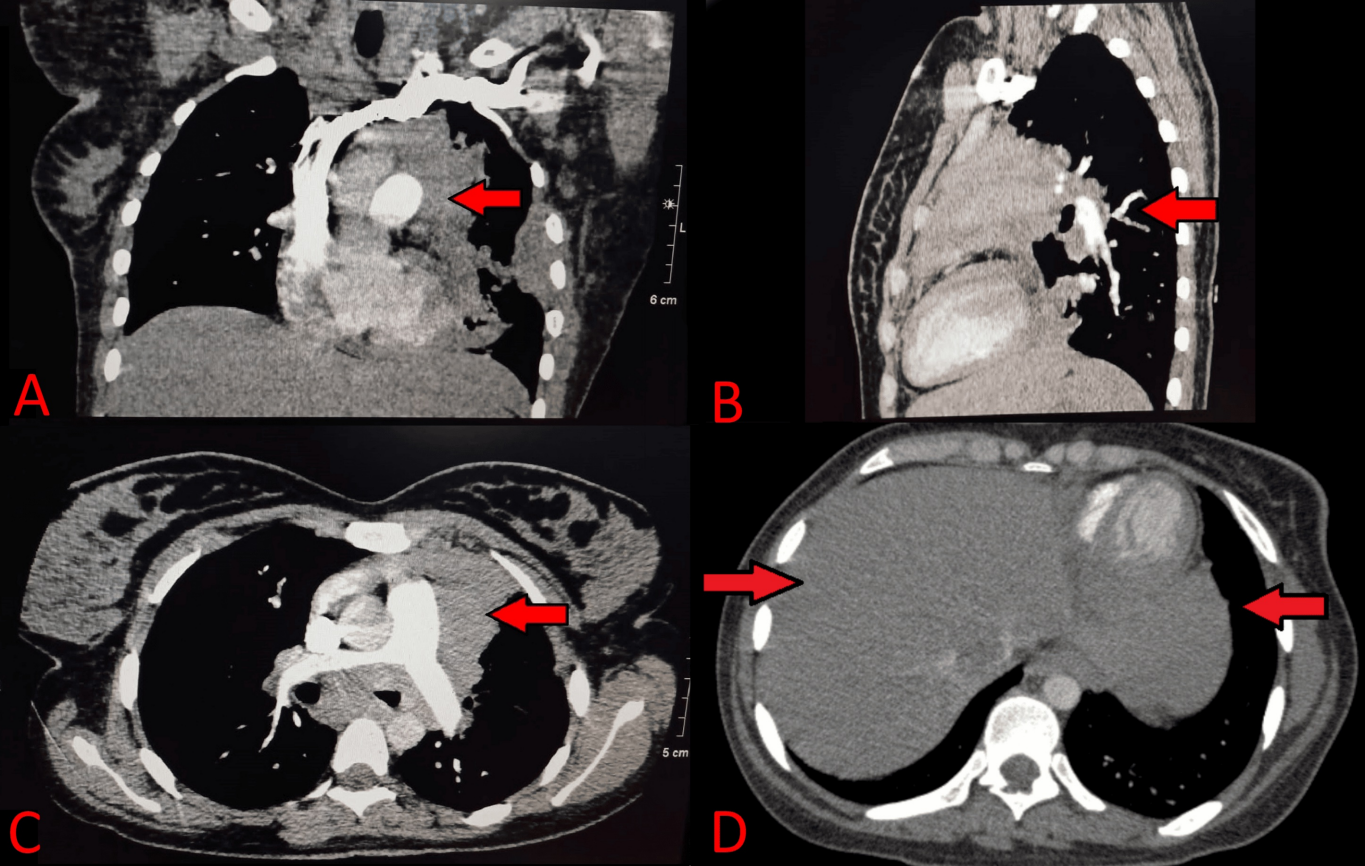
Chest CT scan (A) Coronal view, (B) sagittal view, and (C) axial view of the expanding pulmonary and mediastinal mass. (D) Right lung involvement along with the left.

Thus, a CT pulmonary angiogram was done to visualize the mass further (Figure 4). It revealed a mass lesion extending from the peribronchovascular location in the left upper and right middle lobe extending into the left pleural cavity and along the left hilum to the posterior and middle mediastinum, also invading the left ventricular wall. The possibilities expressed were lymphoreticular disease process or invasive fungal disease.

**Figure 5:**
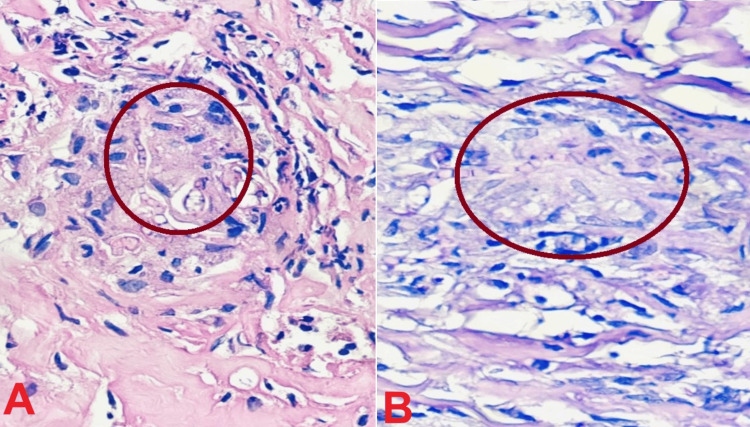
Lung biopsy (A) Biopsy of the lung mass showing intact lung parenchyma with fungal organisms suggestive of *Aspergillus* species. (B) Biopsy of lung mass showing fungal hyphae.

The biopsy of the aforementioned mass lesion was planned under sonographic guidance and microscopy of the lesion showed filamentous fungi suggestive of *Aspergillus* (Figure 5). However, unfortunately, the condition of the patient deteriorated with a drop in her Glasgow coma scale (GCS) rating and the patient expired despite the use of liposomal amphotericin B at a dose of 5 mg/kg/day started one day before her death.

## Discussion

Aspergillosis is an umbrella term for a wide variety of clinical manifestations associated with the *Aspergillus *species, specifically *A. fumigatus, A. niger, *and* A. flavus*. Although it is abundantly present in nature and inhalation of its conidia through the respiratory tract is common, it does not always cause diseases. The disease only occurs with tissue invasion, which happens in immunocompromised states stemming from hematopoietic malignancies, immunodeficiencies, retroviral infections, immunosuppressive drugs, chemo-radiotherapy, transplantation, or stem cell therapies [3].

Usually, initially, *Aspergillus *is confined to the bronchial mucosa and presents with pulmonary symptoms, such as shortness of breath, fever, pleuritic chest pain, hemoptysis, or cough in a neutropenic patient. If transbronchial extension occurs into pulmonary parenchyma, then it is termed invasive, leading to further complications like thrombosis, emboli, and hemorrhagic transformation within the vasculature. No constellation of symptoms is specific for *Aspergillus*, so a neutropenic patient with shortness of breath should be suspected for *Aspergillus*, and on imaging, infiltrates or nodules should point toward the diagnosis as well [4]. The best imaging modality is a CT scan, but even that is not the most sensitive and confirming as radiographic findings vary a lot. Mostly, they appear as segmental areas of consolidation rather than demonstrating the classic nodule progression to cavitation. Tuberculosis is particularly one differential that may present in a similar fashion and have similar findings on imaging [5]. Moreover, other fungi that may cause invasive symptoms may manifest with similar symptoms and findings, and a definitive diagnosis for initiation of therapy is difficult without biopsy and microscopy. CT pulmonary angiography could have some role in differentiating between angioinvasive diseases with infiltrates/nodules/cavitations and non-angioinvasive diseases, but its role is not fully established [6]. Due to its invasive properties, *Aspergillus *could disseminate and have symptoms in other organ systems. It could show up as ring-enhancing lesions in the central nervous system causing seizures or focal neurological signs. Similarly, it could have cutaneous or gastrointestinal spread. There are even cases of it in accidental drowning and instances of spinal cord necrosis caused by disseminated aspergillosis [7].

In recent years, the incidence of *Aspergillus *has increased; nevertheless, it has always been associated with an immunocompromised state and is not considered a disease of the general population. However, there are also cases of it occurring in immunocompetent patients with normal neutrophil levels [8-10], as we saw in our patient who had no predisposing factor of immunocompromise. Her blood work also only showed anemia of chronic disease and eosinophilia, both of which were symptomatically treated until the patient presented with stroke-like symptoms. The only rationale for the anti-helminthic drug was suspicion of parasitemia in the patient, which is a common occurrence in Pakistan due to low standards of lifestyle. As it is not a standard therapy of *Aspergillus,* it continued to extend into the mediastinum and disseminated into the blood, affecting other organs.

Although diagnosing *Aspergillus *has always remained a problem due to non-specific symptoms and radiological findings, there are still ways to confirm it. These include culture with histopathology and microscopy, bronchoalveolar lavage (BAL), galactomannan antigen detection, beta-d-glucan assay, and polymerase chain reaction (PCR). Perhaps, the main issue lies with the fact that raising suspicion about *Aspergillus *and considering it as a differential in immunocompetent patients is not common [11]. This results in late diagnosis and poorer outcomes for patients as seen in this case as well. There are studies where galactomannan enzyme immunoassay was used for pre-emptive therapy. It led to reduced empiric antifungal treatment compared with a culture-based strategy but no change in overall mortality rates [12]. The decision to take biopsies takes too long with vague symptoms, and even then, they are sometimes deferred for risk of complications. This untimely decision-making was seen in the case of this patient as well. Biopsy confirmed aspergillosis as a diagnosis too late, and then in retrospect, the imaging findings and routine blood tests do support the eventual diagnosis in their entirety, like the hyperdense left MCA sign in the CT head of this patient.

Aspergillosis has a very poor prognosis (mortality rate of 30-50%) [13], especially because the general medical condition of the patients that get diagnosed with it means that they have too many comorbidities exacerbating their condition. IA is particularly problematic. These factors can be extrapolated to delineate how a delayed commencement of treatment would automatically have a poorer prognostic value, as was also seen in our patient [14].

The established monotherapy of IA is voriconazole (administered at 6 mg/kg IV twice a day on day 1, followed by 4 mg/kg twice daily for at least seven days, with the option to switch to oral dosing at 200 mg orally twice daily thereafter) [15]. There is a limited role of liposomal amphotericin B in treating invasive *Aspergillus*. In fact, many research papers have shown that voriconazole has established benefits like a greater likelihood of a complete or partial response (53 versus 32 percent), lower mortality rate (29 versus 42 percent), lower rate of severe adverse reactions [16], and better with central nervous system (CNS) involvements of fungus [17]. However, when only a fungal infection is suspected and the exact species is not confirmed, amphotericin B could be a better choice as it gives a greater cover for microbes that may mimic *Aspergillus *symptoms. However, the side effects associated with amphotericin B mean that if used empirically, the decision to switch to a specific therapy should be made in haste and not taken too long. There is also a case to be made for the combination therapy of voriconazole with echinocandin [18]. Nevertheless, by the time the therapy was begun, it was already too late. This tracks as well with the standard timing of the drug administered to combat *Aspergillus*. The minimum duration of therapy ranges from six to 12 weeks. To curb the disease's progress, it is important to diagnose it in time and begin the right therapy immediately [19]. This cannot be achieved through prophylaxis as well, since it too requires voriconazole, a drug that is not generally prescribed or at the forefront of the healthcare provider’s mind when a patient with fever and shortness of breath shows up.

It is important to note that multiple studies have shown that COVID-19 has a complex relationship with aspergillosis, making the prognosis worse; it is possible that this patient could be a case of long-term COVID-19 and acquired aspergillosis conidia, which cascaded into this lethal outcome [20].

Keeping these facts and the case in mind, it seems pertinent to document cases of *Aspergillus *in immunocompetent patients, describing their symptoms, signs, laboratories done, time taken to diagnose, management plans, and final outcomes. Such case reports and case series would allow us to perform more in-depth studies and get a better picture of the causations, associations, and pathophysiology associated with *Aspergillus *and its disease process in immunocompetent individuals.

## Conclusions

Invasive pulmonary aspergillosis is usually considered a disease of the immunocompromised. However, this immunocompetent patient without any risk factors still developed disseminated aspergillosis. Therefore, in patients with a long-standing history of fever and some infectious blood picture, particularly in the setting of eosinophilia with pulmonary involvement, not amenable to other antibiotic and anti-helminthic therapies, it should be considered as a likely potential diagnosis.

We hope that this case report can pave the way for current clinicians to identify and subsequently treat such uncommon cases with decreased durations and morbidities associated with this condition.
